# Effect of acupuncture on the gait disturbance and hemodynamic changes in the prefrontal cortex: a study protocol for a randomized controlled trial

**DOI:** 10.3389/fneur.2024.1444873

**Published:** 2025-01-15

**Authors:** Zhenmei Hong, Shuijing Zhang, Shuqing Zhang, Yuqi Zhao, Xiancong Ye, Xinxin Shu, Yufan Chen, Shuding Yan, Ruijie Ma

**Affiliations:** ^1^Department of Neurology, The Third Affiliated Hospital of Zhejiang Chinese Medical University (Zhongshan Hospital of Zhejiang Province), Hangzhou, China; ^2^Department of Rehabilitation, Zhejiang Rehabilitation Medical Center, Hangzhou, China; ^3^Department of Center for Rehabilitation Assessment and Therapy, Zhejiang Rehabilitation Medical Center, Hangzhou, China; ^4^The Third Clinical Medical College, Zhejiang Chinese Medical University, Hangzhou, China; ^5^Department of Acupuncture and Moxibustion, The Third Affiliated Hospital of Zhejiang Chinese Medical University (Zhongshan Hospital of Zhejiang Province), Hangzhou, China

**Keywords:** acupuncture, gait disturbance, Alzheimer’s disease, study protocol, randomized controlled trial

## Abstract

**Background:**

Alzheimer’s disease (AD) is characterized by cognitive impairment and behavioral impairment. The gait of AD patients is attracting the increasing attention. The aim of this randomized controlled trial (RCT) is to explore the effect of acupuncture on the cognitive function, gait performance, and hemodynamic changes in the prefrontal cortices.

**Methods:**

In this RCT, a total of 108 AD patients will be randomly assigned into acupuncture group or control group for 8 weeks. The primary outcome will be three-dimensional gait analysis and cerebral hemodynamics using functional near-infrared spectroscopy (fNIRS). Secondary outcomes will include Mini-Mental State Examination (MMSE), Montreal Cognitive Assessment (MoCA), and Barthel Index (BI).

**Discussion:**

This trial is expected to explore the effect of acupuncture on cognitive function, gait performance, and hemodynamic changes in the prefrontal cortices for AD patients.

## Introduction

Alzheimer’s disease (AD) is a degenerative disorder of the central nervous system, which is characterized by cognitive impairment and behavioral impairment (1). AD is the most common cause of dementia, and accounts for 60–80% of the cases globally (2). It is estimated that the number of AD patients will exceed 30 million, and about 50% of people over 80 years old will suffer from AD in China by 2050 (3). AD patients not only lose working ability, sociability, self-care ability, and quality of life, but also bring a heavy burden to their families (4).

Non-pharmacological interventions have been reported as the important choices for AD patients (1). Among the non-pharmacological interventions, 78% of patients in China select acupuncture-related treatments (5). Acupuncture has been used clinically in China for many years because of small wound, mild pain, and high security (6, 7). Studies have reported the efficacy of acupuncture in the improvement of cognitive impairment for AD patients (1, 8). Cognitive impairments are often associated with motor disorders, including bradykinesia, rigidity, balance, and gait disorders, which increases the risk of falls (9). The gait of AD patients has gradually attracted attention, and elderly people with AD are twice as likely to suffer from traumatic falls as their peers without AD (10). The effect of acupuncture treatment on the gait performance has been reported in neurodegenerative diseases and brain diseases (11, 12). Pereira et al. found that there were statistically significant differences between acupuncture group and sham-acupuncture group in the gait speed, gait cadence, left–right step length of patients with Parkinson disease, indicating that acupuncture could improve gait in Parkinson disease patients (11). Lou et al. found that step length, gait speed, step frequency, and ground reaction force impulse of stroke patients significantly increased after the acupuncture treatment, indicating that acupuncture could help to improve the gait performance of stroke patients (12). However, the effect of acupuncture on the gait performance has not been reported in AD patients.

Functional near-infrared spectroscopy (fNIRS) is an important tool used to evaluate the neurofunctional activity during walking because it allows to research cerebral hemodynamic activity in an ecological environment without strong immobility constraints (13). A systematic review showed that oxyhemoglobin (HbO_2_) levels within the prefrontal cortices were sensitive to compensation strategies reflecting postural control and gait disorder recovery (13). A study has demonstrated, using fNIRS, that HbO_2_ levels within prefrontal cortices were significantly increased while walking after acupuncture treatment in Parkinson disease patients, and may be sensitive to compensation for restoration of gait disturbance (14). However, fNIRS has not been reported to assess the effects of acupuncture for improving gait disturbance in AD patients.

Therefore, this study aims to assess the clinical effect of acupuncture on gait performance in AD patients and to examine the acupuncture effect on cerebral cortices by identifying hemodynamic changes that occur in the prefrontal cortices using the fNIRS technique.

## Methods

### Study design

**Figure 1:**
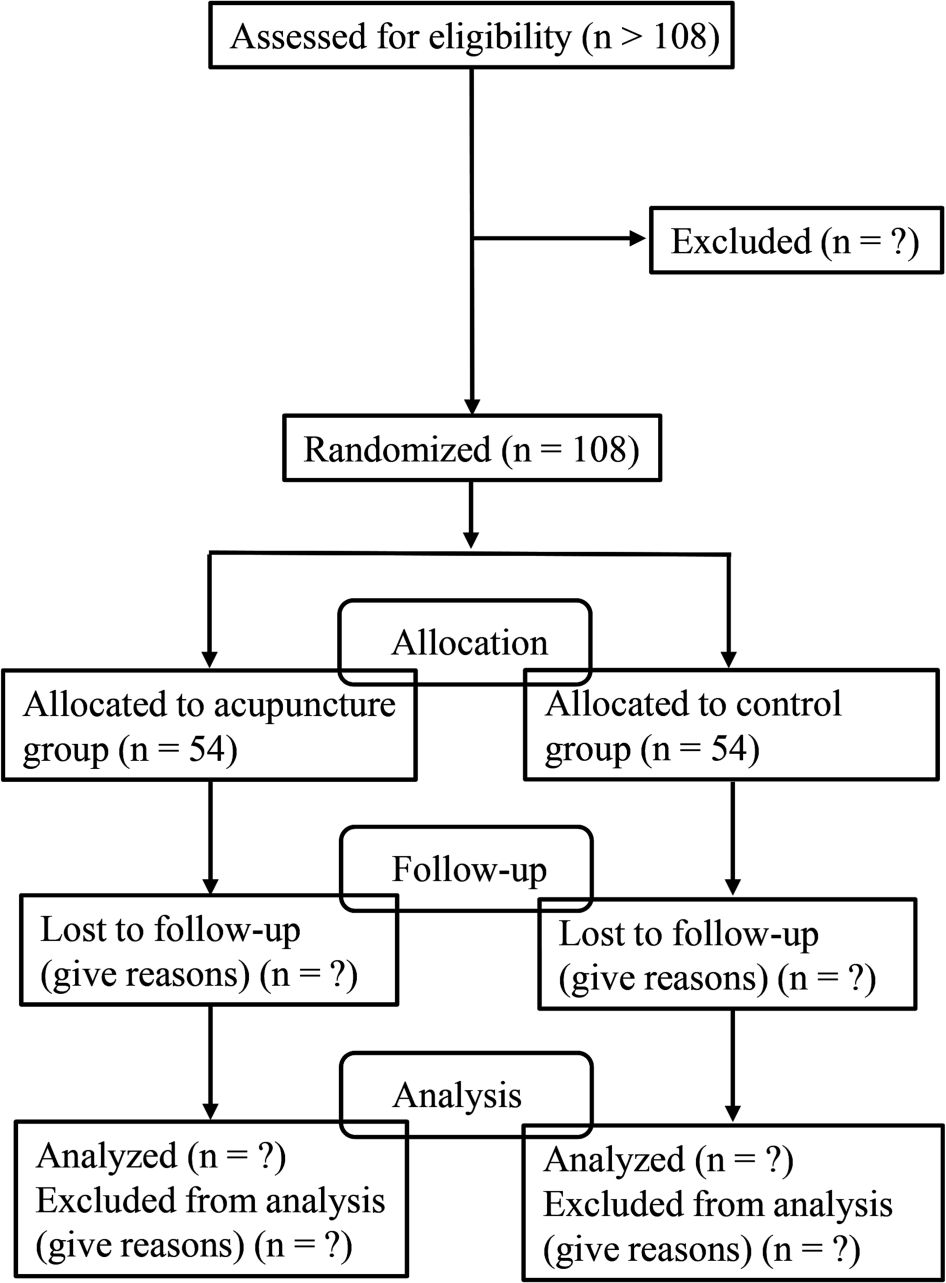
Flowchart of trial process.

This randomized controlled trial (RCT) will be performed in the Third Affiliated Hospital of Zhejiang Chinese Medicine University according to the Declaration of Helsinki. The subjects will be recruited from July 1 to December 31 in 2024 (by the recruitment posters), WeChat (the largest social media platform in China), and from the outpatient and ward of Neurology Department and Neurological Rehabilitation Department. Eligible patients will be randomly assigned to the acupuncture group and control group at the ratio of 1:1. This trial has been approved by the Ethics Committee of the Third Affiliated Hospital of Zhejiang Chinese Medicine University (approval number: ZSLL-KY-2023-015-01), and registered on the ClinicalTrials.gov (NCT06346275).^1^ Informed consent will be signed by each patient (Supplementary material 1). The flowchart is shown in Figure 1, and the timepoint of assessment is shown in Table 1.

**Table 1:** Study schedule of enrollment, intervention, and assessments.

	Enrollment	Allocation	Treatment	
Timepoint		0 day	4-week	8-week
Enrollment				
Eligibility screen	X			
Informed consent	X			
Demographics	X			
Previous medical history	X			
Routine blood	X			
Liver function	X			
Kidney function	X			
Electrocardiograph	X			
Magnetic resonance imaging	X			
Allocation		X		
Interventions				
Acupuncture group			X	X
Control group			X	X
Assessments				
Three-dimensional gait analysis		X	X	X
Cerebral hemodynamics		X	X	X
MMSE		X	X	X
MoCA		X	X	X
BI		X	X	X

MMSE, Mini-Mental State Examination; MoCA, Montreal Cognitive Assessment; BI, Barthel Index.

Patients’ information on demography, diagnosis and treatment history will be recorded, including birth date, gender, ethnicity, age, marital status, occupation, education level, height, weight, medical history, course of illness, treatment history, drug allergy history, and current concomitant medication. Patients are required to complete the laboratory tests, including blood routine, liver and kidney function, electrocardiogram, and magnetic resonance imaging.

### Study population

#### Diagnosis criteria

The diagnostic criteria of AD from Western medicine will be determined according to The National Institute on Aging-Alzheimer’s Association (NIA-AA) (15): (1) cognitive decline that affects daily life and includes damages to two or more cognitive domains; (2) early and most significant cognitive impairment belonging to the past phenotype or non-forgetting phenotype (language disorder, visual spatial disorder, or executive dysfunction); (3) excluding vascular dementia, Lewy body dementia, frontotemporal dementia, primary progressive semantic aphasia, and other active neurological disorders; (4) with biomarkers of brain amyloid-beta (Aβ) protein deposition.

The diagnostic criteria of AD from traditional Chinese medicine will be determined according to Chinese Internal Medicine (16): (1) onset symptoms, main symptoms: decreased ability to remember recent and distant events, cognitive decline (such as ability to judge people, objects, time, location, calculation ability, spatial recognition, language, etc.); accompanying symptoms: character changes such as withdrawn personality, indifferent expression, selfishness and narrowness, stubbornness, irrational euphoria, easy to get excited or angry, decreased understanding, and personality changes such as lack of morality and ethics, and lack of shame; (2) characteristics of onset: hidden onset, slow progression, and long course of disease; (3) syndrome of insufficient marrow-sea: memory loss, loss of recognition and calculation, dull expression, lifeless eyes, low and timid voice or silence all day; slow movement, tinnitus and deafness, withered auricula, hair loss and tooth shaking; red and thin tongue, white tongue moss, and weak pulse.

The dementia stage will be determined using Clinical Dementia Rating (CDR), which assesses cognitive and functional decline from 6 domains: memory, orientation, judgment and problem solving, community affairs, home and hobbies, and personal care (17). The dementia is divided into none (CDR = 0), questionable (CDR = 0.5), mild (CDR = 1), moderate (CDR = 2), and severe level (CDR = 3) (17).

#### Inclusion criteria

The inclusion criteria are as follows:

1. Aged 40–85 years old;
2. Meeting the mentioned-above diagnostic criteria from traditional Chinese medicine and Western medicine;
3. With mild to moderate dementia (CDR = 0.5, 1.0, or 2.0 points);
4. Hachinski Ischemic Scale (HIS) ≤ 4 points;
5. Hamilton Depression Rating Scale (HAMD) < 20 points;
6. Without severe bone and joint diseases and able to walk independently;
7. Able to conduct vision and hearing tests;
8. Volunteering to participate in this trial and signing an informed consent form.

#### Exclusion criteria

The exclusion criteria are as follows:

1. Dementia caused by other systemic or neurological diseases, such as central nervous system infections, post-traumatic dementia, Parkinson’s disease dementia;
2. Suffering from acute illness, upper limb extrapyramidal stiffness, neurological or psychiatric disorders (except cognitive impairment);
3. With medical history that interferes with cognitive function assessment, such as past history of psychiatric drug abuse, drug addiction within the past 5 years, and alcohol abuse;
4. Afraid of acupuncture and cannot accept acupuncture treatment;
5. Currently participating in other clinical trial that affects the outcome evaluation of this trial.

#### Withdrawal criteria

1. Participants themselves requesting to withdraw from the trial;
2. Participants experiencing serious adverse reactions during the trial that are not suitable to continue the trial;
3. Participants experiencing serious complications or the condition worsens that require emergency measures during the trial, and unable to continue the treatment;
4. Patients unable to continue observation due to transferring to another hospital for treatment.

### Randomization and allocation concealment

Prior to the commencement of the trial, we will utilize SPSS version 25.0 software (IBM Corp., Armonk, NY, USA) to generate a random sequence of numbers, allocating participants into two groups (A and B) with a 1:1 ratio. Group A represents the experimental group, while Group B serves as the control group. The random sequence and group allocation information will be securely stored and kept confidential by the principal investigator. We will prepare 108 opaque brown envelopes corresponding to the number of participants to be enrolled. Each envelope will be sequentially numbered on the exterior. Inside each envelope, we will place a sheet containing the corresponding random sequence number and group allocation information. The 108 envelopes will be randomly distributed to the clinical centers involved in the study. Participants will be strictly follow inclusion and exclusion criteria to determine eligibility. Upon confirmation of eligibility, a designated staff member will open the envelopes in ascending order of their numerical identifiers to reveal the group allocation.

### Blinding

Due to the particularity of acupuncture, it is difficult to blind clinical operators and participants. Patients will be informed of the treatment method after receiving grouping information. The efficacy evaluator will be blind. Cognitive function evaluation will be conducted by the researcher who is unaware of the allocation. Liver and kidney function and electrocardiogram testing will be completed by the inspector who is unaware of the allocation. The statistician will be blind when performing statistical analysis. The researchers, clinical operators, and efficacy evaluator are independent.

### Intervention

The control group will receive conventional drug therapy and cognitive rehabilitation therapy. The acupuncture group will receive acupuncture treatment based on the control group. The acupuncture operators are professional acupuncturists who have worked for more than 2 years.

#### Control group

##### Conventional drug therapy

The donepezil tablets (specification: 5 mg × 14 tablets, ZEN Biotechnology Co., Ltd., Chongqing, China) are taken before bedtime (5 mg/time, once a day). Basic treatments are adopted to control blood pressure, blood sugar, blood lipids, and improve brain metabolism.

##### Cognitive rehabilitation therapy

The cognitive training system (JZ-RZ-1020, Extreme Medical Technology, Hangzhou, China) will be used to help develop personalized rehabilitation plans for one-on-one training based on the cognitive function of each patient. The trainings include memory, hand eye reinforcement, attention, reaction. Patients are treated for 30 min each time and 5 times a week for 8 weeks.

#### Acupuncture group

The acupuncture treatment will be conducted based on the control group.

Acupoints: Baihui (GV20), Sishencong (EX-HN1), Fengchi (GB20), Taixi (KI3), Zusanli (ST36), Sanyinjiao (SP6), Neiguan (PC6), and Shenmen (HT7) (Supplementary material 2).

GV20 and EX-HN1: Patients are placed in a suitable position, and local disinfection is performed with 75% alcohol. The 28^#^ stainless-steel needles (Huatuo, Suzhou Medical Instruments Factory, China) will be used in this study with a size of 1.5 inches. When entering the needle, the needle body forms an angle of about 30° with the scalp. The needle tip points forward and enters the GV20, and needle in the EX-HN1 pointed to the GV20 direction. The needle is quickly pierced into the scalp, with a horizontal needling of 0.5 to 0.8 inches. When the resistance under the needle decreases, the needle body is inserted along the subgaleal area in the direction of the acupoint line. When patients achieve the Deqi sensation that characterized by a soreness, numbness, and distention, twisting and replenishing method is used and lasts for 2 min (200 times/min). The needle is left for 6 h, and the twisting and replenishing method is performed every 2 h during the needle retention period. The operation lasts for 1 min each time, for 3 times, until the needle is pulled out.

GB20, KI3, ST36, SP6, PC6, and HT7: Patients are placed in a supine position, followed by acupoints determination and disinfection, and 1.5-inch or 2-inch needle is used. The needle, toward the tip of the nose, is obliquely inserted into GB20 with a depth of 0.8–1.2 inches, and twirling reinforcing-reducing method is used. The needle is obliquely inserted upwards into PC6 with a depth of 1–2 inches, and directly inserted into HT7 with a depth of 0.3–0.5 inches and into KI3 with a depth of 0.5–0.8 inches. The needle, slightly tilted toward the tibia, is directly inserted into ST36 with a depth of 1–2 inches. SP6 is directly needled with a depth of 1–1.5 inches. The twisting and replenishing method is used for limb acupoints, and needles are left for 30 min. The operation when starting the needle is standard to prevent bleeding and hematoma.

To ensure the uniformity of operations, the acupuncture sequence for EX-HN1 is front, back, left, and right. The acupoints on both sides are first punctured on the left side, and then on the right side. Acupuncture treatment is performed once a day (five times a week) and lasts for 8 weeks.

### Follow-up

Both the experimental and control groups will undergo continuous treatment for 8 weeks. Assessments of relevant indicators will be conducted by designated personnel at three time points: before treatment, after 4 weeks, and after 8 weeks of treatment. Additionally, participants will be followed up within 1 month after completing the treatment. Each assessment will take place in a private room, allowing family members to participate in the evaluation process.

### Outcomes

Assessments will be performed before the treatments, at 4 weeks after treatments, and at 8 weeks after treatments.

#### Primary outcomes

Patients undergo three-dimensional gait analysis and fNIRS detection during the walking under three tasks statuses.

Three types of walking task designs: (1) Single gait test: requiring patients to walk in a natural gait within the three-dimensional gait test area without cognitive tasks; (2) Dual task test I: requiring patients to count loudly while walking, increasing the count from 1; (3) Dual task test II: requiring patients to report loudly the names of animals while walking, and try to say as many animal names as possible.

##### Three-dimensional gait analysis

Objective gait quantification under the three different tasks is performed using three-dimensional motion-capture system. British Vicon Optical Motion Capture System (consisting of 6 high-speed infrared cameras) is used to assess the spatiotemporal variables of gait performance, and data are processed by the Vicon Polygon data analysis software. Vicon’s Nexus system detects gait parameters and kinematic parameters, including step length, stride, pace, step width, step frequency, single/double stand phase, turning (time and number of steps required for turning), swing phase.

##### fNIRS

The fNIRS data will be collected using a LIGHTNIRS research-grade portable near-infrared brain function imaging system (manufactured by Shimadzu Corporation, Japan). This system is equipped with 8 light sources and 8 detectors, configured to form 22 channels. It emits light at three wavelengths (780, 805, and 830 nm) and records the raw intensity signals at a sampling frequency of 13.33 Hz. During the gait tasks, we will ensure that the subject’s head is as stationary as possible to minimize movement artifacts. The system will record changes in HbO2 levels in the prefrontal cortex during different walking tasks. The data will be analyzed at three time points: before treatment, after 4 weeks, and after 8 weeks of treatment.

#### Secondary outcomes

##### Mini-mental state examination (MMSE)

MMSE is used to measure cognitive impairment and includes six aspects: time orientation, location orientation, immediate and delayed memory, attention and calculation ability, language, and visual space (18). MMSE is a 30-point questionnaire, and 1 point is assigned for each correct answer. Testing scores are closely related to education level, and the standard for dividing the normal threshold is >17 points for patients with illiteracy, >20 points for patients with primary school, >22 points for patients with secondary school, and > 23 points for patients with junior college (18).

##### Montreal cognitive assessment (MoCA)

MoCA includes eight cognitive domains: attention and concentration, executive function, memory, language, visual space, abstract thinking, computation, and orientation (19). Total score of this scale is 30 points, and patients with a score of ≥26 points are defined as normal (19).

##### Barthel index (BI)

BI is used to assess the physical function status of patients, and the total score ranges from 0 to 100 (20). The functional impairment is divided into mild (> 60 points), moderate (40–60 points), and severe level (≤ 40 points).

#### Adverse events

All patients will be requested to report adverse events, and acupuncturists will record adverse events in detail including date, severity, duration, and measures taken by researchers. The causes are analyzed and causal relationship with the treatment is assessed. Adverse reactions occurring during the trial include dizziness needles, bending needles, and broken needles during acupuncture, adverse physiological reactions during gait walking, and hair heating during the use of fNIRS.

### Data management and quality control

Quality control: (1) Unified technical training for researchers. The content includes familiarizing with the objectives and requirements of this study, mastering relevant diagnostic and treatment standards, random allocation methods, acupuncture methods, and the use of evaluation forms.

(2) Strict record and summarization of this clinical trial. A uniformly printed Case Report Form (CRF) will be adopted, with unified numbering and registration of allocation. Researchers conduct the trial strictly following the project design plan, and carefully and objectively filling out the CRF.

(3) Real time monitoring during the trial. A dedicated quality monitoring team will be established, which will be charged by the principal researcher, who is responsible for verifying whether the researchers have followed the trial protocol and whether the CRF has been filled out in a timely and accurate manner.

(4) Strengthening the compliance. Researchers make patients fully know about the purpose and significance of the study, sign an informed consent form, and provide free treatment and related examinations.

Data management: the CRF is completed in a timely and accurate manner, and then handed over to a dedicated data entry personnel for data storage after inspection by the quality inspector.

### Statistical methods

#### Sample size estimation

Sample size will be calculated from the result of a previous study on the effect of acupuncture on the step frequency at 4 weeks after acupuncture treatment (14). In the study by Jang et al., the step frequency is 115.76 ± 9.59 steps/min in the treatment group and 121.76 ± 10.04 steps/min in the control group (14). In this study, the significance level (*α*) is 0.05, and the desired power (1 − *β*) is 0.80. After calculation by using PASS 11.0 software (NCSS, LLC. Kaysville, Utah), 43 patients are required in each group. Considering a 20% dropout rate, 54 patients are required in each group. Therefore, a total of 108 patients needs to be recruited.

#### Statistical analysis

All data will be analyzed using SPSS 25.0 (IBM, Armonk, NY, USA). Data will undergo the Kolmogorov–Smirnov test to assess normality. The Kolmogorov–Smirnov test is a versatile non-parametric procedure utilized to assess the congruence between an empirical distribution function of a sample and a hypothesized distribution, or to compare two empirical distributions. The test statistic, D, represents the maximum discrepancy between the two cumulative distribution functions. The null hypothesis posits that the distributions are identical. If the calculated D exceeds the critical value at a chosen significance level, the null hypothesis is rejected, implying a significant difference between the distributions. The continuous data in normal distribution will be expressed as mean ± standard deviation (mean ± SD), and differences between two groups are compared using *t* test. The continuous data in skewed distribution will be expressed as medians and interquartile ranges (IQRs), and differences between the two groups are compared using a rank-sum test. The categorical data will be expressed as number (*n*) and percentage (%), and differences between the two groups are compared using the Chi-square. The rank sum test was used for comparison between rank data groups. A *p* < 0.05 (bilateral test) is used as the statistically significant difference in this trial.

## Discussion

Acupuncture, as a complementary and alternative therapy, has been used for the treatment of AD (8). Due to the high safety and few side effects, it is increasingly accepted in the world (21). The main characteristics of AD patients are cognitive impairment and behavioral impairment (1). This trial aims to explore the effect of acupuncture on the cognitive function, gait performance, and hemodynamic changes in the prefrontal cortices.

Acupuncture has neurotrophic and neuroprotective effects (22). During the acupuncture process, patients with cognitive impairment exhibit increased activities in many areas including the temporal lobe, frontal lobe, occipital lobe, and cerebellum posterior lobe compared to the resting state, and most of these areas are related to cognitive impairment (23). Evidence has shown the effective role of acupuncture in the improvement of cognitive impairment in AD patients (1, 8), but RCTs focusing on this topic is insufficient. This RCT will further verify the effect of acupuncture on the cognitive impairment of AD patients.

Gait disorders is a characteristic feature of patients with AD (24). In Parkinson disease and stroke, acupuncture has been reported to improve the gait performance of patients (11, 12). Acupuncture activates the prefrontal and motor cortices, and activation of these areas is associated with a more appropriate motor function (14). In a study, Jang et al. found that the HbO_2_ which indicated brain activity was significantly increased following acupuncture compared to the control group, especially in some channels of the prefrontal cortex (14). The prefrontal cortex has been known to exert an important effect on the balance control (25). The findings by Jang et al. indicated that the activation of prefrontal cortex might improve gait disorders. To determine the effect of acupuncture on the activation of prefrontal cortex in AD patients, we will also explore the changes in prefrontal cortex activity using an fNIRS system.

The trial will observe the effect of acupuncture on the activation of the frontal cortex, and evaluate its role in improving gait performance in AD patients. Compared to previous studies, randomized method is used in this trial the to control confounders. Moreover, the researchers will be trained uniformly, which may decrease the bias. Also, there are several limitations in this trial. First, this is a single-center study. Second, it is difficult to blind acupuncturists and patients due to the nature of acupuncture. Third, the applicability of acupuncture in AD patients with severe dementia remains unclear because our study includes patients with mild to moderate dementia.

## Conclusion

In conclusion, the results of this trial will show the effect of acupuncture on cognitive function, gait performance, and hemodynamic changes in the prefrontal cortices for AD patients.

## Footnotes

^1^ https://clinicaltrials.gov/
