# Supplementary material for: Effect of acupuncture on the gait disturbance and hemodynamic changes in the prefrontal cortex: a study protocol for a randomized controlled trial
Source: Front Neurol. 2025 Jan 15;15:1444873. doi: 10.3389/fneur.2024.1444873 (PMC11777019; doi:10.3389/fneur.2024.1444873)
Supplement: Supplementary file 1 [file Table_1.DOCX]

**Informed Consent Form for Clinical Study**

**Study Title:** Effect of acupuncture on the gait disturbance and hemodynamic changes in the prefrontal cortex: A study protocol for a randomized controlled trial

**Principal Investigator:** [Investigator's Name]
**Affiliate Institution:** [Institution Name]

**Dear Participant,**

Thank you for considering participation in our study. Before you decide whether to participate, please read the following information carefully.

**Purpose of the Study:**
The purpose of this study is to investigate the effects of acupuncture method on the cognitive function, gait performance, and hemodynamic changes in the prefrontal cortices in patients with Alzheimer's Disease (AD).

**Study Procedures:**
If you agree to participate, you will receive the acupuncture treatment. You will be assessed for cognitive function and gait parameters before treatment, after 4 weeks, and after 8 weeks of treatment. Additionally, we will use functional near-infrared spectroscopy (fNIRS) to monitor brain activity during the study.

**Duration of Participation:**
Your participation in this study is expected to last for 8 weeks, including assessments at three time points: before treatment, after 4 weeks, and after 8 weeks.

**Risks and Discomforts:**
Potential risks associated with participation include mild discomfort, such as the sensation of acupuncture or skin irritation. The fNIRS device is safe and poses no long-term effects.

**Compensation expenses related to research related damages:**

If you experience any injury or harm as a result of your participation in this study, you will be compensated for medical treatment, therapy, or other expenses related to the injury, as well as any other compensations as per the study's insurance policy.

**Confidentiality:**
Your personal information will be kept confidential and used solely for research purposes. All data will be anonymized to protect your privacy.

**Voluntary Participation:**
Your participation is entirely voluntary. You have the right to withdraw from the study at any time without facing any penalties or loss of benefits.

**Questions and Concerns:**
If you have any questions or concerns about the study, please contact [Principal Investigator's Name] at [Contact Number].

**Consent to Participate:**
I have read the information above or have had it explained to me. I understand that I can withdraw from the study at any time without any negative consequences. I agree to participate in this study.

**Participant Signature:** ___________________________ Date: _______

**Principal Investigator Signature:** ___________________________ Date: _______

**Contact Information:**
If you have questions after the study is completed, you may contact [Ethics Committee or Regulatory Body Name] at [Contact Number].
