# Supplementary figures and images for: Effect of acupuncture on the gait disturbance and hemodynamic changes in the prefrontal cortex: a study protocol for a randomized controlled trial
Source: Front Neurol. 2025 Jan 15;15:1444873. doi: 10.3389/fneur.2024.1444873 (PMC11777019; doi:10.3389/fneur.2024.1444873)

1. Baihui (GV20)


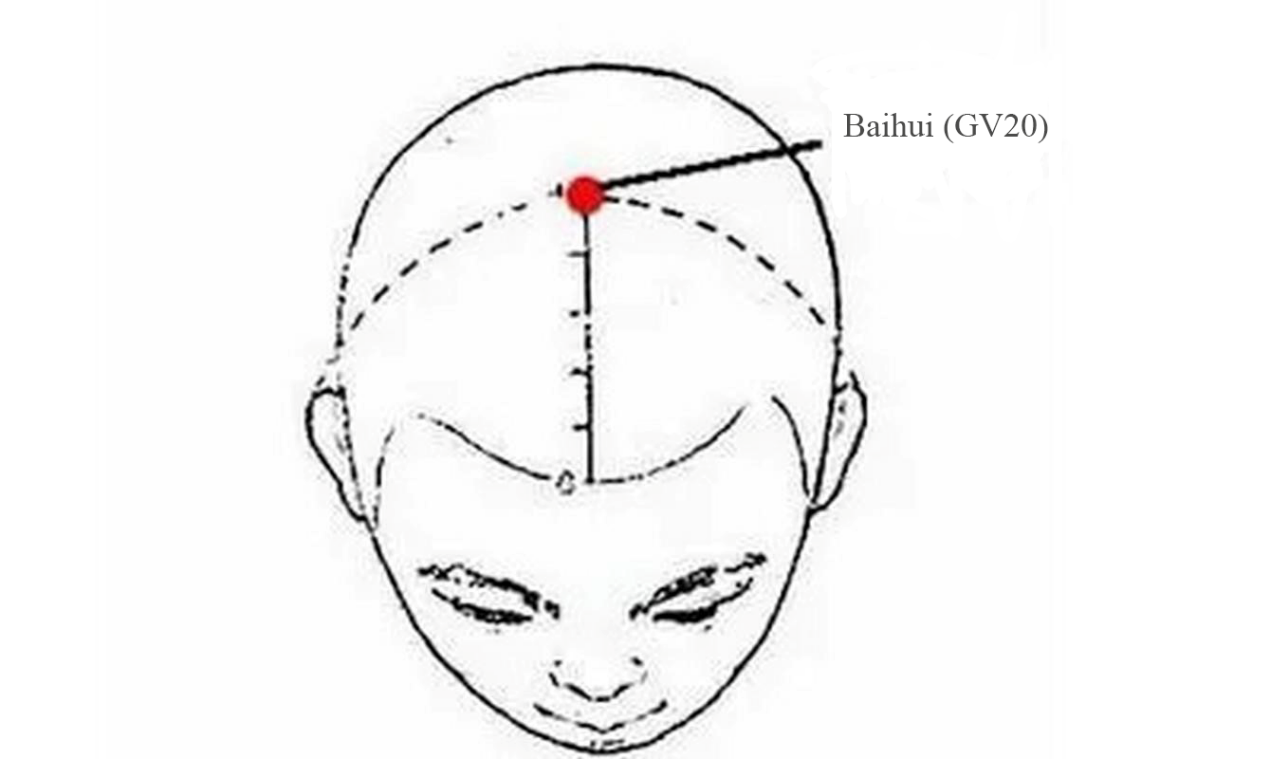


1. Sishencong (EX-HN1)


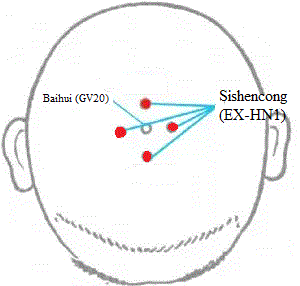


1. Fengchi (GB20)


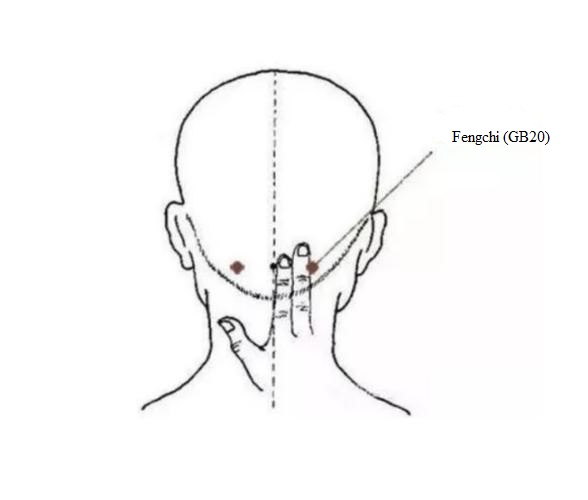


1. Taixi (KI3)


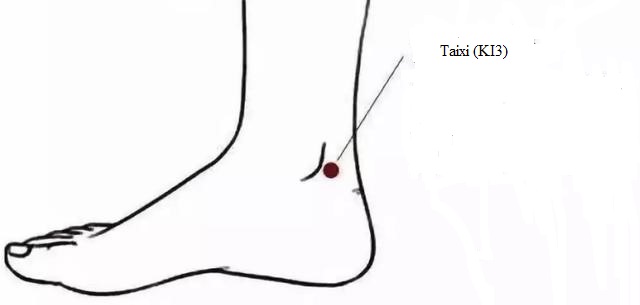


1. Zusanli (ST36)


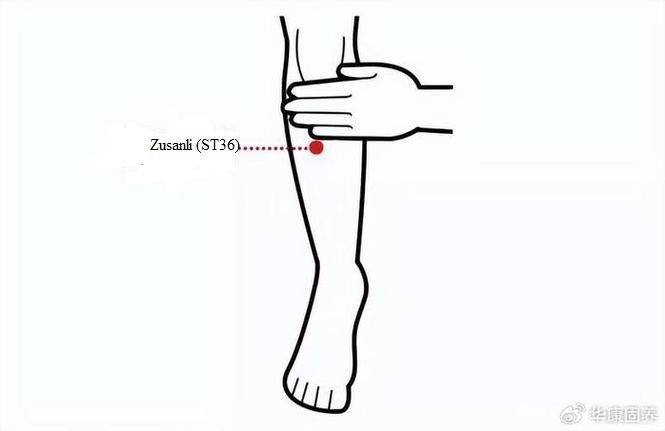


1. Sanyinjiao (SP6)


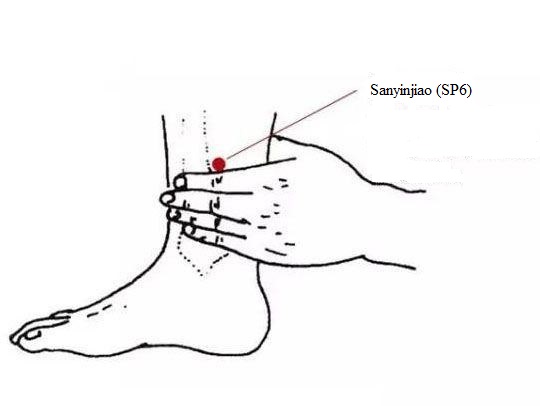


1. Neiguan (PC6)


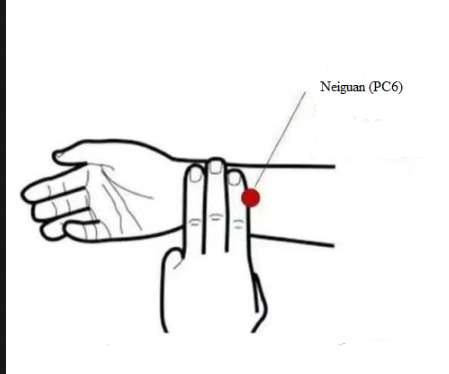


1. Shenmen (HT7)


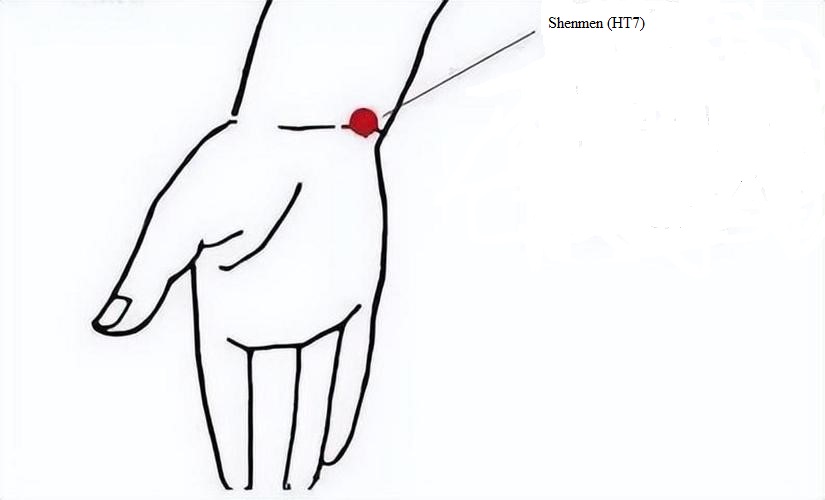

Supplement: Supplementary file 2 [file Table_2.DOCX]
